# Dynamic Changes of Phenolic Composition, Antioxidant Capacity, and Gene Expression in ‘Snow White’ Loquat (*Eriobotrya japonica* Lindl.) Fruit throughout Development and Ripening

**DOI:** 10.3390/ijms25010080

**Published:** 2023-12-20

**Authors:** Kun Zhang, Jiayun Zhou, Panhui Song, Xinyu Li, Xuemei Peng, Yong Huang, Qiaoli Ma, Dong Liang, Qunxian Deng

**Affiliations:** College of Horticulture, Sichuan Agricultural University, Chengdu 611130, China; zhangkun@stu.sicau.edu.cn (K.Z.); 2022305065@stu.sicau.edu.cn (J.Z.); 2022205016@stu.sicau.edu.cn (P.S.); 2022205021@stu.sicau.edu.cn (X.L.); 2022005001@stu.sicau.edu.cn (X.P.); 2023205014@stu.sicau.edu.cn (Y.H.); 2020105004@stu.sicau.edu.cn (Q.M.); 14162@sicau.edu.cn (D.L.)

**Keywords:** white-fleshed loquat, phenolics, antioxidant activity, phenylpropanoid biosynthesis, gene expression

## Abstract

The newly released ‘Snow White’ (SW), a white-fleshed loquat (*Eriobotrya japonica* Lindl.) cultivar, holds promise for commercial production. However, the specifics of the phenolic composition in white-fleshed loquats, along with the antioxidant substances and their regulatory mechanisms, are not yet fully understood. In this study, we examined the dynamic changes in the phenolic compounds, enzyme activities, antioxidant capacity, and gene expression patterns of SW during the key stages of fruit development and ripening. A total of 18 phenolic compounds were identified in SW, with chlorogenic acid, neochlorogenic acid, and coniferyl alcohol being the most predominant. SW demonstrated a stronger antioxidant capacity in the early stages of development, largely due to total phenolics and flavonoids. Neochlorogenic acid may be the most significant antioxidant contributor in loquat. A decline in enzyme activities corresponded with fruit softening. Different genes within a multigene family played distinct roles in the synthesis of phenolics. *C4H1*, *4CL2*, *4CL9*, *HCT*, *CCoAOMT5*, *F5H*, *COMT1*, *CAD6*, and *POD42* were implicated in the regulation of neochlorogenic acid synthesis and accumulation. Consequently, these findings enhance our understanding of phenolic metabolism and offer fresh perspectives on the development of germplasm resources for white-fleshed loquats.

## 1. Introduction

Loquat (*Eriobotrya japonica* Lindl.), originating from China, belongs to the Rosaceae family and is a subtropical evergreen fruit tree [1,2]. Loquat cultivars are categorized into white- and yellow-fleshed varieties [3]. While yellow-fleshed loquats are commonly cultivated, white-fleshed types are less common. White-fleshed fruits are known for their delicious taste and command a higher retail price than yellow-fleshed ones [4]. Consumer interest in white-fleshed loquats is growing, owing to their distinctive nutritional value and flavor.

In recent decades, consumer demand for fruits and vegetables has increased because of their nutritional and health benefits. Loquats can scavenge free radicals and inhibit low-density lipoprotein oxidation, and they are frequently utilized in the food industry to produce juices, fruit wines, and jams [3,5]. The health benefits of loquats are primarily conferred by antioxidants, especially phenolic compounds [6,7]. These phenolic compounds enhance the quality of the fruits, contributing to their sensory and nutritional value [8]. In plants, phenolics are significant secondary metabolites that impart flavor and color to fruits [9,10]. To date, over 10,000 phenolic compounds, including phenols, coumarins, lignans, hydrolysable tannins, phenolic acids, and flavonoids, have been identified in plants [11]. These compounds are primarily synthesized through the phenylpropane metabolic pathway, which is mediated by phenylpropane-related structural genes [12]. For instance, a decrease in the amount of chlorogenic acid—a major phenolic compound in potato—is accompanied by a reduction in the expression level of phenylpropanoid structural genes during plant growth and development [13]. In wheat filling, genes associated with nine phenolic compounds exhibit three distinct expression patterns, suggesting that the transcript levels of genes related to phenolic metabolism correlate with phenolic content [14]. Upregulation of the expression levels of *PAL*, *CHS*, *CHI*, and *DFR* influences the accumulation of phenolic compounds in OE rice (*ZIRc*-overexpressing rice) [15].

Phenolic compounds in loquats have been the focus of numerous studies regarding fruit quality, texture, and antioxidant capacity. For instance, 10 phenolic compounds were identified in the loquat cultivars ‘Mogi’ and ‘Tanaka’, and chlorogenic acid was the specific phenolic compound associated with fruit ripening [16]. The phenolic profiles and antioxidant capacities of six loquat cultivars grown in China were evaluated [17]. Nine phenolic acids were the main phenolic compounds of mature loquat fruits. The ‘Taxiahong’ cultivar contained the highest antioxidant capacity, whereas ‘Taipingbai’ showed the lowest. Qualitative and quantitative analyses of 22 phenolic compounds and 2 terpenoids compounds in loquat peel and flesh revealed that loquat phenolic compounds could attenuate alcohol-induced liver injury [18]. Loquat cultivars grown in Hatay have varied total phenolic contents and high total antioxidant capacity, which are beneficial for human health [19]. To date, few studies have focused on the dynamics of polyphenols and the antioxidant capacity of loquats during fruit development [9,16,17,20]. Certain phenolics, especially phenolic acids and lignin, influence the flavor and texture of the fruit [1].

Phenolic compounds vary according to cultivar, fruit maturity, light, and other factors [21]. Previous studies on loquats have focused on phenolic compounds and cultivars with the highest phenolic content to test for DDPH scavenging ability [9,16,17]. However, little is known about the phenolic compounds, antioxidant substances, and regulatory mechanisms in ‘Snow White’ (SW), a newly selected white-fleshed loquat. Therefore, this study aimed to evaluate the changes in the phenolics and antioxidants in SW during fruit development and ripening. The findings of this study elucidate the physiological processes and molecular mechanisms that govern the changes in the phenolic composition and antioxidant capacity of loquat fruit. These results provide a valuable reference for further exploration of flavor diversity and germplasm enhancement in SW.

## 2. Results and Discussion

### 2.1. Phenotypic Characteristics and Related Indices of Nutritional Quality

**Figure 1:**
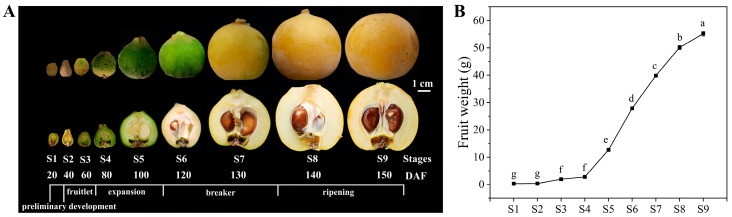
Phenotypic characteristics and fruit weight. (**A**) Fruit phenotype. (**B**) Fruit weight, with lowercase letters indicating significant differences at *p* < 0.05.

The phenotypic characteristics of SW at various developmental stages are depicted in Figure 1A. The transverse sections reveal snow-white flesh. In accordance with our observations and a previous report [22], SW was harvested at nine developmental stages, namely, preliminary development (S1), fruitlet (S2–S3), expansion (S4–S5), breaker (S6–S7), and ripening (S8–S9), which occurred 20, 40, 60, 80, 100, 120, 130, 140, and 150 days after flowering (DAF), respectively. The development of SW fruits spanned an average of approximately 150 days. The fruit development and ripening in SW (Figure 1B) adhered to the typical single sigmoid curve, as was previously described for ‘Dawuxing’ loquat [23]. Loquat fruit ripening is characterized by a multitude of physiological and biochemical transformations, encompassing sugars, acids, phenolic compounds, and other aspects of nutritional quality [24,25].

Nutritional quality is a vital metric for assessing the taste, flavor, and nutritional content of fruit [26]. In the present study, the total soluble solids (TSS), soluble sugars (SS), titratable acidity (TA), vitamin C (Vc), TSS/TA ratio, and SS/TA ratio of loquat pulp are presented in Supplementary Table S1. The TSS of SW reached 13.69%, indicating the high quality of SW [27]. TA and Vc are also crucial for nutritional quality, with Vc being a primary antioxidant in fruits such as oranges and loquats [28]. The TA and Vc content in SW were 0.35% and 1.93 mg/100 g, respectively. These findings suggest that SW has superior nutritional quality and great flavor.

### 2.2. Total Phenolics, Total Flavonoids, and Antioxidant Capacity

**Figure 2:**
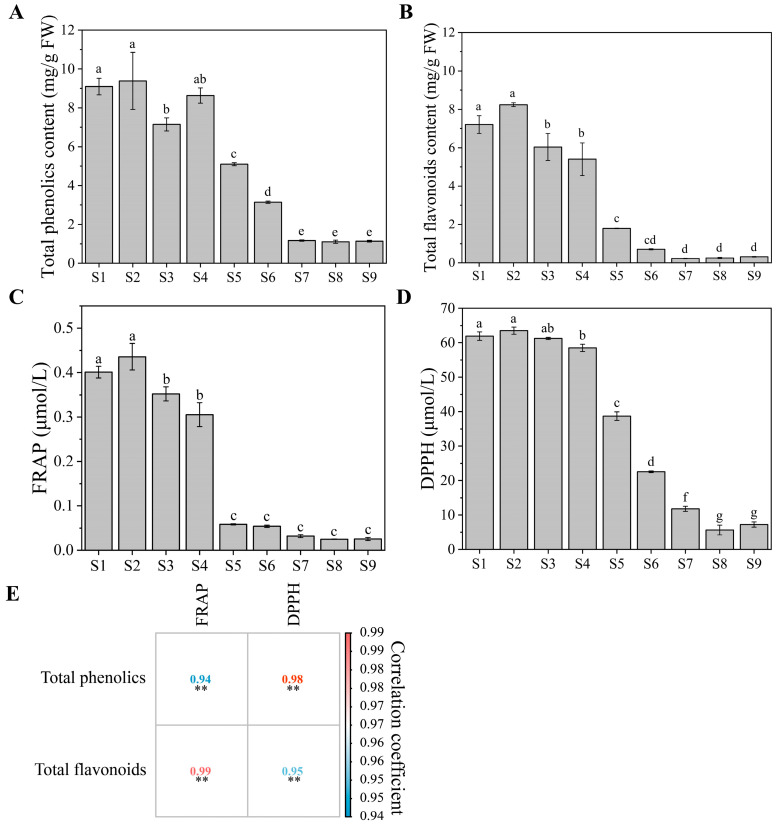
Dynamic changes in total phenolics, total flavonoids, antioxidant capacity, and heatmap analysis. (**A**) Total phenolics content. (**B**) Total flavonoids content. (**C**) Ferric reducing antioxidant power (FRAP) assay. (**D**) 2,2-diphenyl-1-picrylhydrazyl (DPPH) assay. (**E**) Associated heatmap analysis. Different letters indicate significant differences at *p* < 0.05, and the same letter indicates no significant difference. “**” indicates *p* < 0.01.

Loquat is favored by consumers, owing to its delectable taste and abundant dietary antioxidants, along with other bioactive compounds [16]. The contents of total phenolic, total flavonoid, and antioxidant capacity exhibited peak values during S2. They then followed a declining trend to 87.89%, 96.16%, 94.23% (ferric reducing antioxidant power FRAP value), and 88.60% (DPPH value), respectively, by S9 compared with S2 (Figure 2). This reduction in total phenolics, flavonoids, and antioxidant capacity is primarily linked to fruit development and ripening [29]. Elevated phenolic levels confer protection to the fruit against environmental stressors in the early development [30]. As the fruit ripens, these phenolics are consumed through climacteric respiration [31]. At S9, the total phenolic and flavonoid contents were measured to be 1.14 and 0.32 mg/g, respectively (Figure 2A,B). SW displays greater total phenolic and flavonoid contents than yellow-fleshed loquats and other white-fleshed varieties [7,32]. Specifically, ‘Taxiahong’, a yellow-fleshed loquat, presented total phenolic and flavonoid contents of 0.96 mg/g and 0.21 mg/g, respectively. ‘Ninghaibai,’ a white-fleshed variety, had 0.93 mg/g phenolics and 0.15 mg/g flavonoids. Given that phenolics contribute to the flavor profile of fruits [2], these results suggest that SW contains the same bioactive substances as other loquats and is worth promoting.

Phenolics, carotenoids, and Vc are pivotal in scavenging free radicals and mitigating cellular oxidation in loquat. DPPH and FRAP assays are widely employed to assess the antioxidant capacity of loquat fruits [7]. In the present study, the antioxidant capacity, as measured by FRAP and DPPH assays, was highest at S2 and exhibited a decrement throughout the stages (Figure 2C,D). At S2, the antioxidant capacities were recorded at 0.4 μmol/mL (FRAP value) and 63.52 μmol/mL (DPPH value), respectively. The trend of antioxidant capacity was similar to that of total phenolics, with the lowest value obtained at S9. In peach fruits, a significant positive correlation was established between antioxidant capacity and total phenolic content [31]. The acknowledged health benefits of antioxidant compounds underscore the importance of antioxidant capacity as a desirable characteristic in fruit breeding programs. These attributes could be targeted in breeding programs to select superior quality genotypes. In loquat fruits, phenolics are recognized for their significant contributions to antioxidant capacity [32]. Zhou et al. [33] indicated that phenolics are critical antioxidants in loquat. Conversely, Ferreres et al. [9] reported that flavonoids are more contributory to antioxidant properties. A related heatmap was utilized to analyze the correlations among total phenolics, total flavonoids, FRAP, and DPPH. Significant positive correlations, represented in red and blue (*p* < 0.01), are illustrated in Figure 2E. Total phenolics and flavonoids showed highly significant and positive correlations with antioxidant capacity, boasting correlation coefficients exceeding 0.9. These findings highlight the strong interrelationship between total phenolics, flavonoids, and the antioxidant capacity of loquat fruits.

White-fleshed loquats have a delicate flesh and strong flavor, but are difficult to store [2]. Therefore, selecting and breeding new white-fleshed loquats that are suitable for human cultivation and consumption are essential. In the present study, we evaluated the bioactive components, including the classical total phenolics, total flavonoids, Vc, and carotenoids, of the newly selected loquat cultivar, SW. These data will not only help promote the newly selected white-fleshed cultivar, but also serve as a basis for creating a database that will improve the utilization of specific genetic resources in breeding programs [34].

### 2.3. Phenolic Compounds and Content

**Figure 3:**
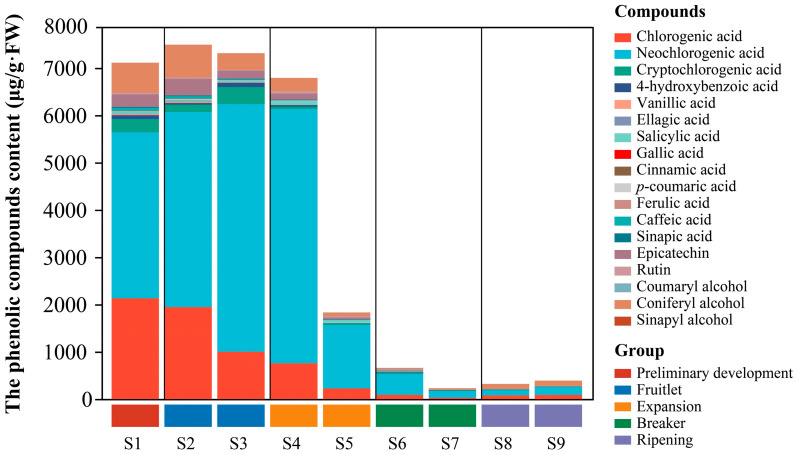
HPLC chromatogram traces and dynamic changes in phenolic compounds.

Phenolic compounds and their content in mature loquat fruits exhibit significant variation among different cultivars [25]. In the present study, 18 phenolic compounds were identified and quantified in SW using high-performance liquid chromatography (HPLC) (Figure 3). The HPLC chromatograms of the phenolic constituents are shown in Supplementary Figure S1. The HPLC chromatograms showed a clear separation of the peaks between different individual phenolic compounds at different UV wavelengths, which indicated that the operational parameters of the HPLC method used in this study were sufficient to effectively separate the phenolic compounds of white-fleshed loquat. The extraction and detection methods were modified, notably for vanillic acid, coniferyl alcohol, and cinnamic acid, which had not been detected in previous analyses of other loquat cultivars [16,17]. The predominant phenolic compounds in SW fruits included chlorogenic acid, neochlorogenic acid, cryptochlorogenic acid, and coniferyl alcohol. In contrast to earlier reports, coniferyl alcohol was also identified as a primary phenolic component in SW [7,16].

At S1–S4, both the chlorogenic and neochlorogenic acid contents were found to be high, echoing findings from studies on different loquat fruits [7,16]. Chlorogenic acid, a member of the phenylpropanoids, presented high levels at S1, peaking at 2129.65 μg/g fresh weight (FW), and decreased to 88 μg/g FW at S9. Neochlorogenic acid was the most prevalent phenolic compound, registering concentrations of 3511.25 μg/g FW at S1 and 163.97 μg/g FW at S9. The content of neochlorogenic acid sharply declined at S4–S5, which may be due to its conversion into downstream metabolites [11]. Coniferyl alcohol, at 107.98 μg/g FW, ranked as the second-most abundant phenolic compound following neochlorogenic acid at S9. An increase in neochlorogenic acid and coniferyl alcohol at S8 and S9 could indicate physiological markers of loquat fruit ripening [16].

By contrast, only nine phenolic compounds were detected at harvest. Previous research has indicated that the phenolic composition in young fruits is more diverse than that in mature loquat fruits [25]. The phenolic content across different loquat varieties is not uniform, and the antioxidant activity attributed to various phenolic fractions also varies. Active phenolics such as chlorogenic acid, neochlorogenic acid, cryptochlorogenic acid, and pinacol have been recognized as key antioxidants in loquats. The findings of the present study on antioxidant content and capacity suggest that neochlorogenic acid is the most significant antioxidant in loquat. This study provides novel insights into the dynamic profile of phenolics in the newly released SW cultivar.

Tulipani et al. [8] concluded that genetic background plays a key role in the chemical and nutritional composition of plants and found significant differences in total antioxidant capacity and phenolic acid composition between genotypes. During breeding, phenolic compounds and antioxidant capacity are thoroughly evaluated using accurate quantitative methods. This can provide breeders with specific information on the traits of a new variety and help understand its genetic background. Cantin et al. [35] evaluated the antioxidant capacity and phenolic compounds of different nectarine-breeding offspring and selected peach genotypes with enhanced antioxidant capacity to produce fruits with health-promoting properties. Therefore, high-quality parents were selected for breeding work to ensure that the parents in the breeding program had good antioxidant properties and rich phenolic fractions. This helps to pass on these superior traits to the progeny.

### 2.4. Boxplot Diagram and Principal Component Analysis (PCA)

**Figure 4:**
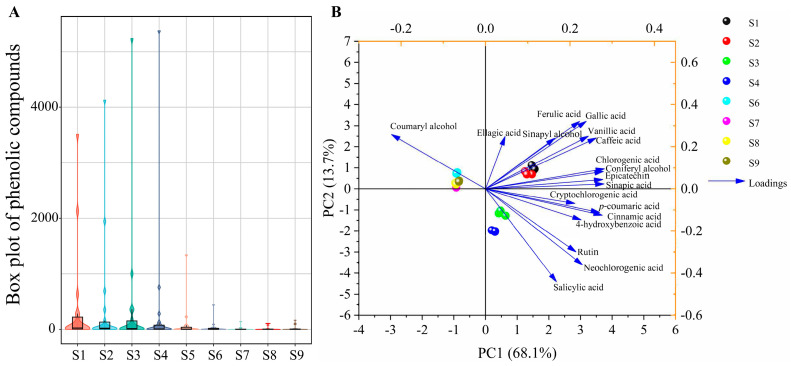
Boxplot diagram and PCA of phenolic profiles. (**A**) Boxplot representation. (**B**) PCA biplot illustrating the distribution of phenolics.

The boxplot diagram illustrates the distribution of phenolic content (Figure 4A). At S1–S4, the distribution of phenolic content gradually increased, peaking at S4, where the concentration of individual phenolic components was highest, before subsequently decreasing. To delve deeper into the impact of these variations on the fruit quality of SW, PCA was conducted on phenolic attributes (Figure 4B). The data points representing different phenolic compounds were dispersed across distinct areas. All the phenolic compounds were accounted for on the first principal component (PC1, which explained 68.1% of the variance) and on the second principal component (PC2, which explained 13.7% of the variance). SW at S1–S5 could be differentiated by PC1 from that at S6–S9 based on phenolic compounds. PC1 positively correlated with most phenolic compounds and negatively correlated with coumaryl alcohol. S1 and S2 were positioned in the first quadrant, strongly associated with vanillic acid, chlorogenic acid, and caffeic acid. All phenolic compounds contributed to the explanation on PC1 (68.1%), whereas some, including epicatechin, coumaric acid, and chlorogenic acid, exhibited negative loadings on PC2 (13.7%). Phenolic compounds such as chlorogenic acid, coniferyl alcohol, epicatechin, sinapyl alcohol, cinnamic acid, and *p*-coumaric acid had significant loadings on PC1, indicating that S1 and S2 substantially influenced the phenolic content.

### 2.5. Phenolic Metabolism-Related Enzyme Activities

**Figure 5:**
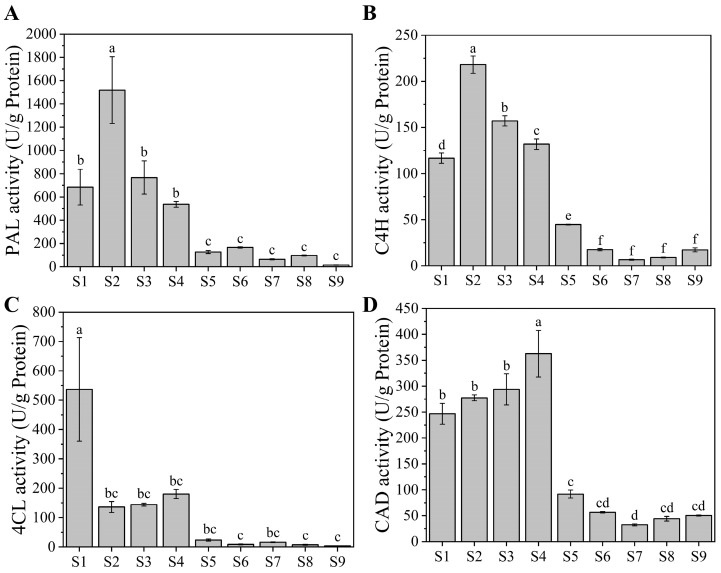
Activities of enzymes related to phenolic metabolism. (**A**) PAL activity. (**B**) C4H activity. (**C**) 4CL activity. (**D**) CAD activity. Different letters indicate significant differences at *p* < 0.05, and the same letter indicates no significant difference.

The activities of enzymes involved in phenylpropanoid synthesis, including phenylalanine ammonia-lyase (PAL), cinnamate 4-hydroxylase (C4H), 4-coumarate-CoA ligase (4CL), and cinnamyl alcohol dehydrogenase (CAD), are associated with the biosynthesis of phenolic compounds [36]. PAL is the initial enzyme in the monolignol biosynthetic pathway, catalyzing the deamination of L-phenylalanine to trans-cinnamic acid. This compound is a precursor for various phenylpropanoids such as flavonoids, coumarins, and lignins [37]. C4H catalyzes the conversion of trans-cinnamic acid to *p*-coumaric acid, followed by the transformation of the latter into 4-coumaroyl-CoA by 4CL [38]. CAD activity primarily affects phenolic compounds by altering lignin content [37]. In the present study, the dynamic trends of PAL, C4H, 4CL, and CAD activities mirrored those of total phenolics, total flavonoids, and antioxidant capacity (Figure 5). Notably, the activities of PAL and C4H were elevated at S2. This could be due to the buildup of phenolics during the pre-growth and developmental periods of the fruit [39]. As the fruit matured, the activity of PAL gradually decreased. Previous research has indicated that the decline in PAL activity correlates with reduced lignin content in the cell walls of loquat fruits, contributing to fruit softening [40]. CAD activity was notably highest at S4, which coincides with the accumulation pattern of neochlorogenic acid. In pigeon peas, genes related to phenylpropane metabolism, including CAD, play a role in synthesizing chlorogenic acid, caffeic acid, ferulic acid, and *p*-coumaric acid [41]. Thus, CAD might be implicated in the regulation of neochlorogenic acid synthesis.

### 2.6. Gene Expression Patterns of Phenolic Metabolism

**Figure 6:**
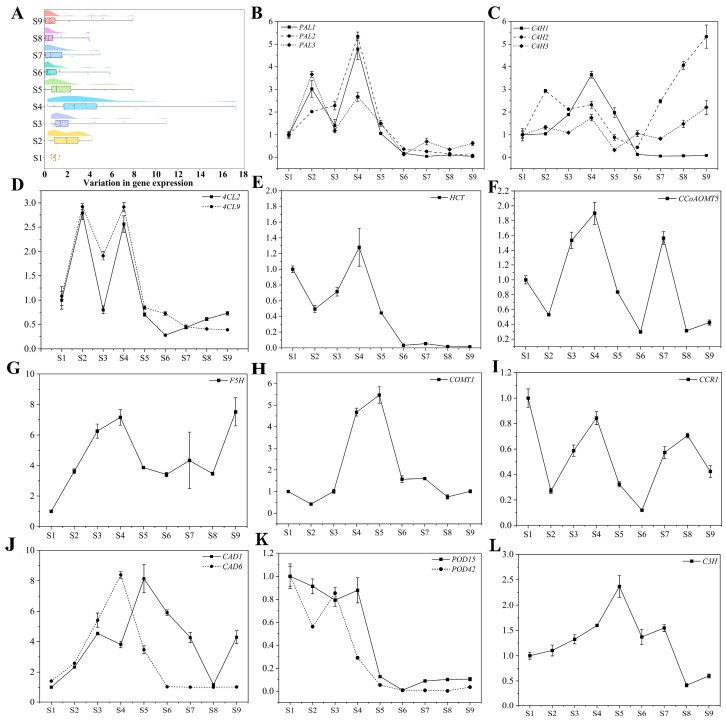
Gene expression trends and genes involved in phenolic metabolism. (**A**) Gene expression trends. (**B**–**L**) Expression of genes involved in phenolic metabolism, including PAL; C4H; 4CL; HCT: hydroxycinnamoyl-CoA shikimate/quinate hydroxycinnamoyl transferase; CCoAOMT: caffeoyl-CoA O-methyltransferase; F5H: ferulate-5-hydroxylase; COMT: caffeic acid 3-O-methyltransferase; CCR: cinnamoyl-CoA reductase; CAD; POD: peroxidase; C3H: coumaric acid-3-hydroxylase.

The gene expression trends and relative gene expression levels of accumulating phenolics were analyzed to further elucidate the molecular mechanisms underlying the phenolic metabolism in SW (Figure 6). The accumulation of phenolic compounds in fruits is strongly associated with genes involved in phenylpropanoid biosynthesis [37,42,43]. To the best of our knowledge, no systematic study on the genes regulating phenolic metabolism in white-fleshed loquats has been reported to date.

Phenolic acids in plants are predominantly synthesized through the phenylpropane biosynthetic pathway. *PAL*, *C4H*, *4CL*, *HCT*, *F5H*, *COMT*, *CAD*, and *CCR* mediate the synthesis of various phenolic acid compounds and their derivatives [12]. Corresponding to the trend in phenolic content distribution, the expression of all genes showed the most significant variation at S4 (Figure 6A). The expression pattern of *PAL* exhibited two modest peaks at S2 and S4. The dual peaks of PAL activity reported in strawberry fruits, with one peak during the early green fruit stage, have been associated with the synthesis of phenolics in the early stages of fruit development [39]. *C4H1*, *4CL2*, *4CL9*, *HCT*, *CCoAOMT5*, *F5H*, *COMT1*, *CAD6*, and *POD42* were expressed at higher levels at S4, which coincides with rapid fruit expansion and peak accumulation of neochlorogenic acid, suggesting their involvement in the regulation of neochlorogenic acid synthesis and accumulation. Similar to findings in the loquat cultivar ‘Changhong’, cold treatment elevated the transcript levels of *EjPAL1*, *Ej4CL2*, *EjCCoAOMT*, *EjHCT*, and *EjCCR*, which was concurrent with the accumulation of chlorogenic, neochlorogenic, and cryptochlorogenic acids [44].

*F5H* and *CCR* have been identified as key genes in the deposition of phenolic acids, including caffeic acid, ferulic acid, and pinacol, in dates [45]. Notably, while most of the genes were expressed at lower levels in the later stages, *F5H* exhibited a higher transcription level at S9 than at other stages, suggesting its role in phenolic accumulation not only during pre-fruit development, but also in flavonoid synthesis in the later stages of fruit development [46,47]. In *Arabidopsis*, *CCR1*-deficient mutants exhibited increased levels of ferulic and malic acids [48]. In the present study, the content of ferulic acid increased by 48.35% and 31.98% at S2 and S9, respectively, indicating that the downregulation of *CCR1* expression during both periods was associated with ferulic acid accumulation. C3H and COMT enzymes catalyze the synthesis of ferulic acid from *p*-coumaric acid [49]. The changes in *C3H* and *COMT1* expression followed a similar trend, although ferulic acid accumulation was not observed at S5. This might reflect the distinct roles of various genes within the multigene family in phenolics synthesis [14]. In summary, the phenolic metabolism of SW is co-regulated by multiple genes.

### 2.7. Analysis of Relationships

**Figure 7:**
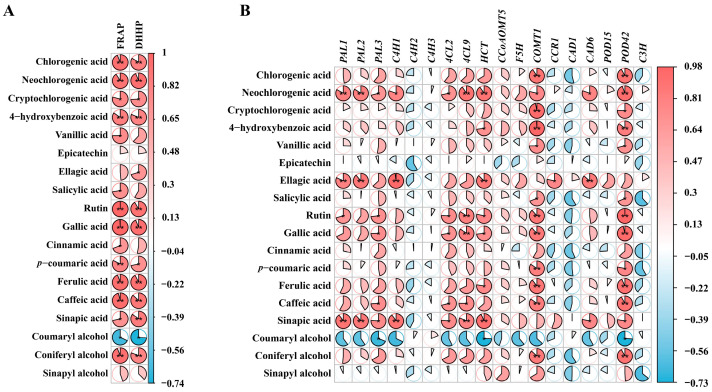
Correlation analysis of phenolic compounds with antioxidant capacity and genes. (**A**) Correlation analysis of phenolic compounds with antioxidant capacity. (**B**) Correlation analysis of phenolic compounds with phenolics metabolism genes. The values and color intensities are proportional to the correlation coefficient. Blue indicates negative correlation and red indicates positive correlation (*p* < 0.05). “*” indicates *p* < 0.05 and “**” indicates *p* < 0.01.

The correlation analysis between phenolic compounds, antioxidant capacity, and genes has revealed more detailed information (Figure 7). In the present study, most phenolic compounds were significantly positively correlated (*p* ≤ 0.05) with antioxidant capacity, except for coumaryl alcohol, which exhibited a significant negative correlation (*p* ≤ 0.05) (Figure 7A). This finding is in line with the result of previous studies that phenolic compounds positively correlate with FRAP and DPPH [20,50]. Additionally, significant positive correlations were observed between phenolic compounds and the genes *PAL*, *4CL*, *HCT*, *COMT1*, *CAD6*, and *POD42* (Figure 7B). *COMT1* and *POD42* showed significant positive correlations with the accumulation of 12 and 13 phenolic compounds, respectively, including chlorogenic acid, neochlorogenic acid, cryptochlorogenic acid, and coniferyl alcohol. Thus, the dynamic changes in phenolics in SW could be ascribed to the coordinated regulation of multiple genes at distinct stages of phenolic acid metabolism.

**Figure 8:**
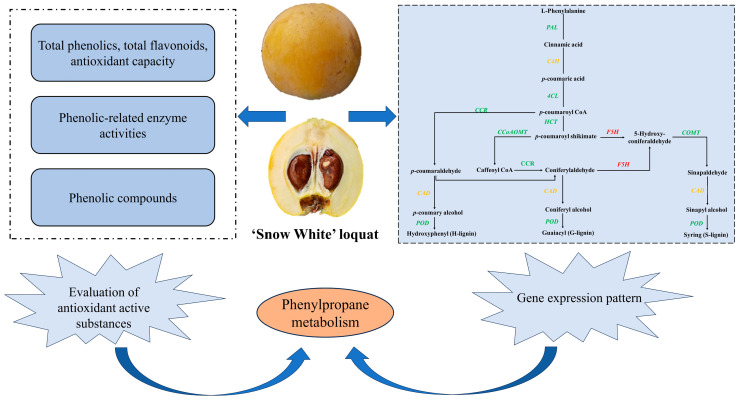
Dynamic changes in phenolics, antioxidant system, and gene expression of loquat fruit. The red indicates an increase, the green indicates a decrease, and yellow indicates both an increase and a decrease.

In this study, we explored the dynamic changes in phenolic composition and the antioxidant capacity of SW during growth and development and elucidated the underlying regulatory mechanisms at the physiological and transcriptional levels (Figure 8). The results indicated that SW possessed a higher antioxidant capacity and phenolic content than other cultivars. Particularly, it contained coniferyl alcohol, which had not been detected in previously reported white-fleshed loquats. Eighteen phenolic compounds were identified throughout the growth and development of the white-fleshed loquat. Neochlorogenic acid, coniferyl alcohol, and chlorogenic acid were most prominent at S9. Total flavonoids, total phenolics, and neochlorogenic acid were significant contributors to the antioxidant capacity of SW. *C4H1*, *4CL2*, *4CL9*, *HCT*, *CCoAOMT5*, *F5H*, *COMT1*, *CAD6*, and *POD42* participated in the regulation of neochlorogenic acid synthesis and its accumulation. The downregulation of *CCR1* expression at S2 and S9 correlated with the accumulation of ferulic acid. In summary, SW is a newly released white-fleshed loquat cultivar that is characterized by a rich composition of phenolic compounds and antioxidants, and its synthesis and accumulation are directed by phenylpropanoid pathway genes.

## 3. Materials and Methods

### 3.1. Plant Material Growth Conditions and Sample Preparation

This study was conducted using five-year-old SW-bearing loquat trees from the loquat germplasm resource nursery of Sichuan Agricultural University, located at Shimian County, Ya’an City, Sichuan Province, China (29°18′55″ N–29°18′56″ N, 102°32′16″ E). SW was grafted on DWX rootstock with a spacing of 4 m between rows and 4 m between plants. At least 30 fruits with the same size and color were collected from three plants of each cultivar, and three biological replications were set up. All collected fruit samples were chopped and frozen in liquid nitrogen and then stored at −80 °C until use. Throughout this experiment, all plants were managed according to standard horticultural practices.

### 3.2. Determination of Fruit Weight, and Nutritional Quality

Fruit weight was measured using an electronic balance, and total soluble solid (TSS) content was determined using a digital refractometer (Atago, Tokyo, Japan). The soluble sugar (SS) content was determined using the anthrone colorimetric method [51]. Titratable acidity (TA) content was determined by titration with NaOH and expressed as % malic acid [52]. The vitamin C (Vc) content was determined using the AOAC method and expressed as mg/100 g fresh weight [32].

### 3.3. Determination of Total Phenolics Content, Flavonoids Content, and Antioxidant Capacity

The total phenolic and flavonoid contents were determined following a previous method with slight modifications [5]. The total phenolic content was determined using the Folin–Ciocalteu method. Briefly, 2 g of frozen pulp was ground with 5 mL of 80% methanol (*v*/*v*) containing 2% formic acid and centrifuged at 12,000× *g* for 10 min at 4 °C. Then, the supernatant was mixed with 1 mL of Folin–Ciocalteu reagent and 0.8 mL of 1 mol/L sodium carbonate. The mixed solution was incubated at 30 °C for 1 h and the absorbance was recorded at 765 nm. The content was expressed as gallic acid equivalent (μg GAE/g·FW). Total flavonoids were determined as follows. Two grams of loquat fruits were homogenized with 10 mL of 80% cold acetone and centrifuged at 12,000× *g* for 20 min at 4 °C. The resulting extract (2.0 mL) was combined with 2.0 mL of 3% AlCl_3_ and 1.0 mL of 70% (*v*/*v*) ethanol in a reaction mixture. The mixture was then incubated at room temperature for 10 min. After incubation, the absorbance was measured at 510 nm, and the content was expressed as rutin equivalent (μg rutin/g·FW). The DPPH and FRAP assays were determined according to a previous method [53]. The results were expressed as μmol/L water-soluble vitamin E (Trolox) equivalent antioxidant capacity.

### 3.4. Determination of Enzyme Activities

The activities of PAL, C4H, and 4CL were measured following a previously described method with minor modifications [36].

PAL activity was determined as follows. Briefly, 0.5 g of pulp was ground to powder under liquid nitrogen, added with 5 mL of 50 mmol/L sodium borate buffer (pH = 8.8) containing 20 mmol/L *β*-mercaptoethanol, and then centrifuged at 4 °C for 30 min at 8000 r/min. The supernatant was collected, and 3 mL of 50 mmol/L boric acid buffer (pH = 8.8), 0.5 mL of 20 mmol/L L-phenylalanine solution, 0.5 mL of enzyme extract, and 0.5 mL of inactivated enzyme solution were added to two test tubes separately. The mixture was placed in a 37 °C water bath for 60 min, and then the reaction was terminated by adding 0.1 mL of 6 mol/L hydrochloric acid solution. One unit of PAL activity was defined as the amount of enzyme that caused an increase in absorbance of 0.01 at 290 nm/min.

C4H activity was measured as follows. Briefly, 0.5 g of pulp was ground with 3.0 mL of extract solution containing 50.0 mmol/L Tris-HCL (pH = 8.9), 15 mmol/L *β*-mercaptoethanol, 4.0 mmol/L magnesium chloride, 5.0 mmol/L vitamin C, 10 μmol/L leupeptin hemisulfate salt, and 10% glycerol. After 1 h of reaction, the supernatant was collected by centrifugation at 8000 r/min for 30 min at 4 °C. A reaction mixture containing 0.8 mL of crude enzyme solution, 2.2 mL of the reaction solution, 2.0 μmol/L cinnamic acid, 2.0 μmol/L sodium salt of oxidized coenzyme II, and 5.0 μmol/L disodium glucose hexakisphosphate was incubated at 30 °C for 30 min. One unit of C4H activity was defined as the amount of enzyme that caused an increase in absorbance of 0.01 at 340 nm/min.

4CL activity was determined as follows. Briefly, 0.5 g of pulp was ground with 3 mL of 50 mmol L Tris-HCL (pH = 8.0) and then centrifuged at 8000 r/min for 4 min at 4 °C. The supernatant was collected to measure 4CL activity. The reaction mixture contained 0.4 mL of crude enzyme solution and 2.6 mL of reaction solution (containing 50.0 mmol/L Tris-HCL (pH = 8.0), 5.0 μmol/L *p*-coumaric acid, 50.0 μmol/L adenosine triphosphate, 1.0 μmol/L thioglycocoenzyme A, and 15.0 μmol/L magnesium sulfate heptahydrate). The mixture was reacted at 40 °C for 30 min. One unit of 4CL activity was defined as the amount of enzyme that caused an increase in absorbance of 0.01 at 333 nm/min, respectively.

CAD activity was determined as previously described by Cai et al. [54] with slight modifications. Briefly, 0.5 g of pulp powder was extracted using 3 mL of Tris:HCl buffer (200 mM, pH = 7.5). The reaction mixture consisted of 100 mmol/L Tris:HCl (pH = 8.8), 20 mmol/L coniferyl alcohol, 5 mmol/L NADP, and 50 μL of extraction solution. The reaction solution was placed in a water bath at 37 °C for 30 min. The reaction was terminated by adding 0.5 mL of 1 mol/L HCl. One unit of CAD activity was defined as the amount of enzyme that caused an increase in absorbance of 0.01 at 340 nm/min.

### 3.5. Determination of Phenolic Compounds and Content

Phenolic compound standards, including chlorogenic acid, neochlorogenic acid, cryptochlorogenic acid, 4-hydroxybenzoic acid, vanillic acid, epicatechin, ellagic acid, salicylic acid, rutin, gallic acid, cinnamic acid, *p*-coumaric acid, ferulic acid, caffeic acid, sinapic acid, coumaryl alcohol, coniferyl alcohol, and sinapyl alcohol, were purchased from Beijing Solarbio Science & Technology Co. Ltd. (Beijing, China). Ultrapure water was prepared using a Millipore water purification system (Millipore Corporation, Burlington, MA, USA) with a 0.22 μm filtration layer. Chemicals were purchased from Chengdu Hao Bo You Technology Co., Ltd. (Chengdu, China).

Phenolic compounds were extracted using previously described methods with slight modifications [5]. Briefly, 1 g of frozen tissue was extracted with 3 mL of 80% (*v*/*v*) cold methanol under light-proof conditions. The mixture was extracted in a sonicator for 30 min and then centrifuged at 12,000× *g* for 10 min at 4 °C. Subsequently, the supernatant was purified using the C18 Sep-Pak cartridge (Sep-Pak Vac 6cc, Waters, Milford, MA, USA) and filtered through a 0.22 µm membrane. Phenolic compounds were analyzed using an HPLC system (Agilent, Santa Clara, CA, USA) equipped with a UV photodiode array detection and a Comatex C18 column (4.6 × 250 mm, 5 μm; CoMetro, Princeton, NJ, USA). The operating temperature of the column was maintained at 30 °C, the injection volume was 20 μL, and the detection wavelengths of the UV photodiode array were at 280 and 320 nm, respectively. Mobile phase A was methanol and mobile phase B was ultrapure water containing 0.1% phosphoric acid, with gradient elution at a flow rate of 1 mL/min. The gradient elution program was as follows: 5%–71% A (0–22 min), 71%–5% A (22–30 min), and 5% A (30–35 min). The quantification of individual phenolic compounds was analyzed based on the calibration curve and the result was expressed as μg/g fresh weight. The different phenolic compounds were effectively separated in this study and the standard curve equations for the phenolic compounds are shown in Supplementary Table S2.

### 3.6. Analyze Quantitative Real-Time PCR (qRT-PCR)

The qRT-PCR primers of the selected genes were designed based on transcriptome sequences using Primer Premier 5 software (Premier Biosoft, Palo Alto, CA, USA). The detailed primer sequences are shown in Supplementary Table S3. qRT-PCR was performed on a CFX96 Real-Time System C1000 Thermal Cycler (Bio-Rad, Hercules, CA, USA) using the ArtiCanCEO SYBR qPCR Mix (Tsingke, Beijing, China) according to the instructions. Relative gene expression was normalized to the reference gene Actin. Gene expression levels were calculated using the 2^−ΔΔ^ CT method.

### 3.7. Statistical Analysis

All data, except transcriptomic, were subjected to Tukey’s honestly significant difference test to assess differences between means at a significance level of 0.05. SPSS 22.0 (IBM, Chicago, IL, USA) and data were expressed as the means ± standard deviation (SD). Principal component analysis (PCA) was conducted using OriginPro2021 (OriginLab, Northampton, MA, USA). The association was determined using Pearson’s correlation coefficient (*p* ≤ 0.05 or 0.01).

## Data Availability

The data and materials supporting the conclusions of this study are included within the article.

## Supplementary Materials

The following supporting information can be downloaded at: https://www.mdpi.com/article/10.3390/ijms25010080/s1, Figure S1: The HPLC chromatograms of the phenolic constituents; Table S1: Relevant nutritional quality indicators of SW loquat; Table S2: Authentic standard phenolic compounds used in the HPLC analysis and equations of standard curves; Table S3: Primers for real-time PCR.

## References

[1] Chi Z., Liu X., Wen S., Wang Y., Lv W., Guo Q., Xia Y., Jing D., Liang G. (2023). Integrated metabolomic profiling and transcriptome analysis of fruit quality and ripeness in early-maturing seedless triploid loquat. Sci. Hortic..

[2] Zou S., Wu J., Shahid M.Q., He Y., Lin S., Liu Z., Yang X. (2020). Identification of key taste components in loquat using widely targeted metabolomics. Food Chem..

[3] Jing D., Liu X., He Q., Dang J., Hu R., Xia Y., Wu D., Wang S., Zhang Y., Xia Q. (2023). Genome assembly of wild loquat (*Eriobotrya japonica*) and resequencing provide new insights into the genomic evolution and fruit domestication in loquat. Hortic. Res..

[4] Yellow D. (2016). DNA markers based on *PSY* genes can differentiate yellow- and white-fleshed loquats. J. Am. Pomol. Soc..

[5] Wang L., Shao S., Madebo M.P., Hou Y., Zheng Y., Jin P. (2020). Effect of nano-SiO_2_ packing on postharvest quality and antioxidant capacity of loquat fruit under ambient temperature storage. Food Chem..

[6] Sultan M.Z., de la Rosa L.A., Alvarez-Parrilla E., González-Aguilar G.A. (2017). Loquat (*Eriobotrya japonica* Lindl.). Fruit and Vegetable Phytochemicals: Chemistry and Human Health.

[7] Xu H.X., Chen J.W. (2011). Commercial quality, major bioactive compound content and antioxidant capacity of 12 cultivars of loquat (*Eriobotrya japonica* Lindl.) fruits. J. Sci. Food Agric..

[8] Tulipani S., Mezzetti B., Capocasa F., Bompadre S., Beekwilder J., De Vos C.R., Capanoglu E., Bovy A., Battino M. (2008). Antioxidants, phenolic compounds, and nutritional quality of different strawberry genotypes. J. Agric. Food Chem..

[9] Ferreres F., Gomes D., Valentão P., Gonçalves R., Pio R., Chagas E.A., Seabra R.M., Andrade P.B. (2009). Improved loquat (*Eriobotrya japonica* Lindl.) cultivars: Variation of phenolics and antioxidative potential. Food Chem..

[10] Giada M., Morales-González J.A. (2013). Food phenolic compounds: Main classes, sources and their antioxidant power. Oxidative Stress and Chronic Degenerative Diseases-A Role for Antioxidants.

[11] Liang D., Deng H., Deng Q., Lin L., Lv X., Wang J., Wang Z., Xiong B., Zhao X., Xia H. (2020). Dynamic changes of phenolic compounds and their associated gene expression profiles occurring during fruit development and ripening of the Donghong kiwifruit. J. Agric. Food Chem..

[12] Lin W., Li Y., Lu Q., Lu H., Li J. (2020). Combined analysis of the metabolome and transcriptome identified candidate genes involved in phenolic acid biosynthesis in the leaves of *Cyclocarya paliurus*. Int. J. Mol. Sci..

[13] Payyavula R.S., Navarre D.A., Kuhl J., Pantoja A. (2013). Developmental effects on phenolic, flavonol, anthocyanin, and carotenoid metabolites and gene expression in potatoes. J. Agric. Food Chem..

[14] Ma D., Li Y., Zhang J., Wang C., Qin H., Ding H., Xie Y., Guo T. (2016). Accumulation of phenolic compounds and expression profiles of phenolic acid biosynthesis-related genes in developing grains of white, purple, and red wheat. Front. Plant Sci..

[15] Qi Q., Li W., Yu X., Zhang B., Shang L., Xie Y., Li Y., Ding A., Shi J., Dou Y. (2023). Genome-wide analysis, metabolomics, and transcriptomics reveal the molecular basis of *ZlRc* overexpression in promoting phenolic compound accumulation in rice seeds. Food Front..

[16] Ding C., Chachin K., Ueda Y., Imahori Y., Wang C.Y. (2001). Metabolism of phenolic compounds during loquat fruit development. J. Agric. Food Chem..

[17] Xu H., Li X., Chen J. (2014). Comparison of phenolic compound contents and antioxidant capacities of loquat (*Eriobotrya japonica* Lindl.) fruits. Food Sci. Biotechnol..

[18] Yan Q., Chen Y., Wu M., Yang H., Cao J., Sun C., Wang Y. (2023). Phenolics and terpenoids profiling in diverse loquat fruit varieties and systematic assessment of their mitigation of alcohol-induced oxidative stress. Antioxidants.

[19] Polat A.A., Çalişkan O., Serce S., Saraçoğlu O., Kaya C., Özgen M. (2010). Determining total phenolic content and total antioxidant capacity of loquat cultivars grown in Hatay. Pharmacogn. Mag..

[20] Zhang W., Zhao X., Sun C., Li X., Chen K. (2015). Phenolic composition from different loquat (*Eriobotrya japonica* Lindl.) cultivars grown in China and their antioxidant properties. Molecules.

[21] Tomás-Barberán F.A., Espín J.C. (2001). Phenolic compounds and related enzymes as determinants of quality in fruits and vegetables. J. Sci. Food Agric..

[22] Fu X., Kong W., Peng G., Zhou J., Azam M., Xu C., Grierson D., Chen K. (2012). Plastid structure and carotenogenic gene expression in red-and white-fleshed loquat (*Eriobotrya japonica*) fruits. J. Exp. Bot..

[23] Deng H., Li X., Wang Y., Ma Q., Zeng Y., Xiang Y., Chen M., Zhang H., Xia H., Liang D. (2023). Organic acid accumulation and associated dynamic changes in enzyme activity and gene expression during fruit development and ripening of common loquat and its interspecific hybrid. Foods.

[24] Hu C., Gao X., Dou K., Zhu C., Zhou Y., Hu Z. (2023). Physiological and metabolic changes in tamarillo (*Solanum betaceum*) during fruit ripening. Molecules.

[25] Cai J., Chen T., Zhang Z., Li B., Qin G., Tian S. (2019). Metabolic dynamics during loquat fruit ripening and postharvest technologies. Front. Plant Sci..

[26] Kader A.A. (2008). Flavor quality of fruits and vegetables. J. Sci. Food Agric..

[27] Tian S., Qin G., Li B., Yahia E.M. (2011). Loquat (*Eriobotrya japonica* L.). Postharvest Biology and Technology of Tropical and Subtropical Fruits.

[28] Del Caro A., Piga A., Vacca V., Agabbio M. (2004). Changes of flavonoids, vitamin C and antioxidant capacity in minimally processed citrus segments and juices during storage. Food Chem..

[29] Siriamornpun S., Kaewseejan N. (2017). Quality, bioactive compounds and antioxidant capacity of selected climacteric fruits with relation to their maturity. Sci. Hortic..

[30] Fait A., Hanhineva K., Beleggia R., Dai N., Rogachev I., Nikiforova V.J., Fernie A.R., Aharoni A. (2008). Reconfiguration of the achene and receptacle metabolic networks during strawberry fruit development. Plant Physiol..

[31] Li Y., Li L., Zhang X., Tian J., Yan J., Guo L., Wang Y., Song L., Yu X. (2023). Differences in total phenolics, antioxidant activity and metabolic characteristics in peach fruits at different stages of ripening. LWT.

[32] Ercisli S., Gozlekci S., Sengul M., Hegedus A., Tepe S. (2012). Some physicochemical characteristics, bioactive content and antioxidant capacity of loquat (*Eriobotrya japonica* (Thunb.) Lindl.) fruits from Turkey. Sci. Hortic..

[33] Zhou C.H., Li X., Xu C.J., Sun C.D., Chen K.S. (2012). Hydrophilic and lipophilic antioxidant activity of loquat fruits. J. Food Biochem..

[34] Gentile C., Reig C., Corona O., Todaro A., Mazzaglia A., Perrone A., Gianguzzi G., Agusti M., Farina V. (2016). Pomological traits, sensory profile and nutraceutical properties of nine cultivars of loquat (*Eriobotrya japonica* Lindl.) fruits grown in Mediterranean area. Plant Foods Hum. Nutr..

[35] Cantin C.M., Moreno M.A., Gogorcena Y. (2009). Evaluation of the antioxidant capacity, phenolic compounds, and vitamin C content of different peach and nectarine [*Prunus persica* (L.) Batsch] breeding progenies. J. Agric. Food Chem..

[36] Wang L., Shan T., Xie B., Ling C., Shao S., Jin P., Zheng Y. (2019). Glycine betaine reduces chilling injury in peach fruit by enhancing phenolic and sugar metabolisms. Food Chem..

[37] Singh R., Rastogi S., Dwivedi U.N. (2010). Phenylpropanoid metabolism in ripening fruits. Compr. Rev. Food Sci. Food Saf..

[38] Douglas C.J. (1996). Phenylpropanoid metabolism and lignin biosynthesis: From weeds to trees. Trends Plant Sci..

[39] Cheng G.W., Breen P.J. (1991). Activity of phenylalanine ammonia-lyase (PAL) and concentrations of anthocyanins and phenolics in developing strawberry fruit. J. Am. Soc. Hortic. Sci..

[40] Shah H.M.S., Khan A.S., Singh Z., Ayyub S. (2023). Postharvest Biology and Technology of Loquat (*Eriobotrya japonica* Lindl.). Foods.

[41] Hussain K., Jaweed T.H., Kamble A.C. (2023). Modulation of phenylpropanoid and lignin biosynthetic pathway is crucial for conferring resistance in pigeon pea against Fusarium wilt. Gene.

[42] Sharma A., Shahzad B., Rehman A., Bhardwaj R., Landi M., Zheng B. (2019). Response of phenylpropanoid pathway and the role of polyphenols in plants under abiotic stress. Molecules.

[43] Inostroza-Blancheteau C., Reyes-Díaz M., Arellano A., Latsague M., Acevedo P., Loyola R., Arce-Johnson P., Alberdi M. (2014). Effects of UV-B radiation on anatomical characteristics, phenolic compounds and gene expression of the phenylpropanoid pathway in highbush blueberry leaves. Plant Physiol. Biochem..

[44] Hou Y., Liu Y., Zhao L., Zhao Y., Wu Z., Zheng Y., Jin P. (2023). *EjCML19* and *EjWRKY7* synergistically function in calcium chloride-alleviated chilling injury of loquat fruit. Postharvest Biol. Technol..

[45] Zhang Q., Wang L., Wang Z., Zhang R., Liu P., Liu M., Liu Z., Zhao Z., Wang L., Chen X. (2021). The regulation of cell wall lignification and lignin biosynthesis during pigmentation of winter jujube. Hortic. Res..

[46] Zhao X., Zhao Y., Gou M., Liu C.J. (2023). Tissue-preferential recruitment of electron transfer chains for cytochrome P450-catalyzed phenolic biosynthesis. Sci. Adv..

[47] Li X., Bonawitz N.D., Weng J.K., Chapple C. (2010). The growth reduction associated with repressed lignin biosynthesis in Arabidopsis thaliana is independent of flavonoids. Plant Cell.

[48] Mir Derikvand M., Sierra J.B., Ruel K., Pollet B., Do C.T., Thévenin J., Buffard D., Jouanin L., Lapierre C. (2008). Redirection of the phenylpropanoid pathway to feruloyl malate in *Arabidopsis* mutants deficient for cinnamoyl-CoA reductase 1. Planta.

[49] Ma Y., Wang P., Wang M., Sun M., Gu Z., Yang R. (2019). GABA mediates phenolic compounds accumulation and the antioxidant system enhancement in germinated hulless barley under NaCl stress. Food Chem..

[50] Lafarga T., Villaró S., Bobo G., Simó J., Aguiló-Aguayo I. (2019). Bioaccessibility and antioxidant activity of phenolic compounds in cooked pulses. Int. J. Food Sci. Technol..

[51] Ebell L. (1969). Variation in total soluble sugars of conifer tissues with method of analysis. Phytochemistry.

[52] Song H., Yuan W., Jin P., Wang W., Wang X., Yang L., Zhang Y. (2016). Effects of chitosan/nano-silica on postharvest quality and antioxidant capacity of loquat fruit during cold storage. Postharvest Biol. Technol..

[53] Rumpf J., Burger R., Schulze M. (2023). Statistical evaluation of DPPH, ABTS, FRAP, and Folin-Ciocalteu assays to assess the antioxidant capacity of lignins. Int. J. Biol. Macromol..

[54] Cai C., Xu C., Li X., Ferguson I., Chen K. (2006). Accumulation of lignin in relation to change in activities of lignification enzymes in loquat fruit flesh after harvest. Postharvest Biol. Technol..

