# Supplementary material for: Dynamic Changes of Phenolic Composition, Antioxidant Capacity, and Gene Expression in ‘Snow White’ Loquat (Eriobotrya japonica Lindl.) Fruit throughout Development and Ripening
Source: Int J Mol Sci. 2023 Dec 20;25(1):80. doi: 10.3390/ijms25010080 (PMC10779426; doi:10.3390/ijms25010080)
Supplement: Supplementary file 1 [file ijms-25-00080-s001.zip › ijms-2742282-supplementary.pdf]

Supplementary Tables

Table S1. Relevant nutritional quality indicators of SW loquat

Stage	Soluble sugars (%)	Total soluble solids (%)	Titratable acidity (%)	Vitamin C (mg/100g FW)	SSC/TA	TSS/TA
S9	13.69±0.27	11.29±0.24	0.35±0.006	1.93±0.17	39.5	32.57

Table S2. Authentic standard phenolic compounds used in the HPLC analysis and equations of standard curves

Phenolic compounds	Linear regression equation	Correlation coefficient
Chlorogenic acid	$y = 25.947x + 510.86$	0.9954
Neochlorogenic acid	$y = 16.445x + 141.66$	0.9981
Cryptochlorogenic acid	$y = 9.2498x + 49.317$	0.9998
4-hydroxybenzoic acid	$y = 11.676x + 48.378$	0.9995
Vanillic acid	$y = 16.120x + 52.804$	0.9998
Epicatechin	$y = 6.9703x + 47.318$	0.9991
Ellagic acid	$y = 10.753x + 45.311$	0.9985
Salicylic acid	$y = 5.9264x + 8.3776$	0.9992
Rutin	$y = 14.243x + 19.973$	0.9999
Gallic acid	$y = 23.535x + 51.863$	0.9994
Cinnamic acid	$y = 26.625x + 475.59$	0.9967
p-coumaric acid	$y = 80.188x - 333.47$	0.9997
Ferulic acid	$y = 70.277x + 188.01$	0.9981
Caffeic acid	$y = 61.044x + 260.12$	0.9977
Sinapic acid	$y =82.507x + 670.13$	0.9992
Coumaryl alcohol	$y = 0.2428x + 4.0361$	0.9942
Coniferyl alcohol	$y = 2.9723x + 19.880$	0.9997
Sinapyl alcohol	$y = 49.318x + 99.461$	0.9996

Table S3. Primers for real-time PCR

Gene	Forward primer sequence (5'- 3')	Reverse primer sequence (5'- 3')
Actin	AATGGAACTGGAATGGTCAAGGC	TGCCAGATCTTCTCCATGTCATCCCA
PAL1	TGCTACAGAATCAGGCCACA	GCATGGAGTGACGTTGTTGT
PAL2	TTTGCCTACATTGATGAC	CATTCTTCTCACTCTCAC
PAL3	CAGTGCTACATATCCTCTAA	CATCTCCTTCTCACCATT
C4H1	CCCGATATTAAGGCCGTTTT	GCTTCGAGGATGTGGTCAAT
C4H2	CTCCTTCTTCCCCACATGAA	GGACTCCTCCTCCAAAAACC
C4H3	AGTCGAAGGTGGAGGCTAAC	ACTGTCCACCTTTCTCCGAG
4CL2	TGGACGGAGAGAATCCAAAC	GCTCCAGCAGCGTACCTATC
4CL9	AGTCTCACAGGGATATGGGATG	GACATCAGCCTTCCATTAGCTC
HCT	CTACAGGCCGAATCCAAACG	CCGCCTCAACAAACAGAACA
CCoAOMT5	ATGGAGAGGAGCAGCAAACT	ATTCAGGCTCCCTTGGGTAC
F5H	ACGACAAGTCCTCCAAGTCCAA	AGTCCACTTCGTCCCTAACAGA
COMT1	GGCTGCCCACTCTACCATTA	CTCCTCCAACATGCTTCACA

CCR1	AGAGAAAGGGGTGGACTTGG	TGCACATAGGCCTGAACTGA
CAD1	TCTGGAGTTCTCTCGCCGTA	TGCCATGCTTGTTCCTTGGA
CAD6	TGGCTCAGACGACTCCAAAT	TGGTCACATCGTTGATCCCA
POD15	ATGAATCATGCCCGAATGCC	TCGCAACCATTGACAAAGCA
POD42	GAGAGTCTCTGCCCCAAAGT	GCTGTGGTGGAGTCTCTTCT
C3H	GGCTAGCATTTGGGAAACGT	TGTCTCTGTTTGCTCCGTGT

Supplementary Figure

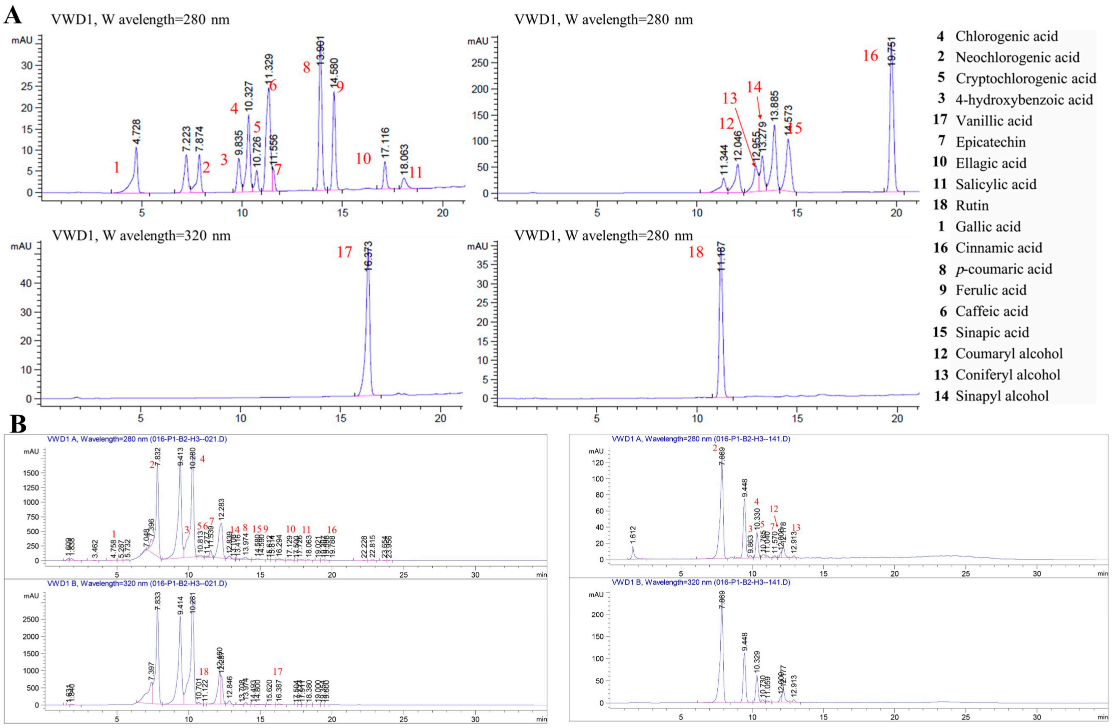

Figure S1. The HPLC chromatograms of the phenolic constituents. (A) Chromatogram of polyphenol standard compounds. (B) Chromatograms of methanol-water (80:20, v/v) fruit extracts made at 280 and 320 nm wavelengths, respectively. Peak numbers 1−18 refer to chlorogenic acid, neochlorogenic acid, cryptochlorogenic acid, 4-hydroxybenzoic acid, vanillic acid, epicatechin, ellagic acid, salicylic acid, rutin, gallic acid, cinnamic acid, *p*-coumaric acid, ferulic acid, caffeic acid, sinapic acid, coumaryl alcohol, coniferyl al-cohol, and sinapyl alcohol, respectively.
